# Evaluation of disinfection methods for personal protective equipment (PPE) items for reuse during a pandemic

**DOI:** 10.1371/journal.pone.0287664

**Published:** 2023-07-27

**Authors:** John Archer, Anne Mikelonis, Barbara Wyrzykowska-Ceradini, Eric Morris, Jonathan Sawyer, Timothy Chamberlain, Ahmed Abdel-Hady, Mariela Monge, Abderrahmane Touati

**Affiliations:** 1 U.S. Environmental Protection Agency, Office of Research and Development, Center for Environmental Solutions and Emergency Response, Homeland Security and Materials Management Division, Research Triangle Park, North Carolina, United States of America; 2 Jacobs Technology Inc., Research Triangle Park, North Carolina, United States of America; 3 Science Systems and Applications Inc., Research Triangle Park, North Carolina, United States of America; 4 CSS Inc., Research Triangle Park, North Carolina, United States of America; Fiocruz Mato Grosso do Sul, BRAZIL

## Abstract

The COVID-19 pandemic resulted in many supply chain issues, including crippling of essential personal protective equipment (PPE) needed for high-risk occupations such as those in healthcare. As a result of these supply chain issues, unprecedented crisis capacity strategies were implemented to divert PPE items such as filtering facepiece respirators (FFRs, namely N95s) to those who needed them most for protection. Large-scale methods for decontamination were used throughout the world to preserve these items and provided for their extended use. The general public also adopted the use of non-specialized protective equipment such as face coverings. So, the need for cleaning, decontamination, or disinfection of these items in addition to normal clothing items became a necessary reality. Some items could be laundered, but other items were not appropriate for washing/drying. To fill research gaps in small-scale, non-commercial cleaning and disinfection, this bench-scale research was conducted using small coupons (swatches) of multiple PPE/barrier protection materials inoculated with virus (non-pathogenic bacteriophages Phi6 and MS2) and tested against a range of decontamination methods including bleach-, alcohol- and quaternary ammonium compound (QAC)-based liquid sprays, as well as low concentration hydrogen peroxide vapor (LCHPV) and bench-scale laundering. In general, non-porous items were easier to disinfect than porous items, and the enveloped virus Phi6 was overall easier to inactivate than MS2. Multiple disinfection methods were shown to be effective in reducing viral loads from PPE coupons, though only laundering and LCHPV were effective for all materials tested that were inoculated with Phi6. Applications of this and follow-on full-scale research are to provide simple effective cleaning/disinfection methods for use during the current and future pandemics.

## Introduction

Once reserved for and used primarily by healthcare, scientific, and other industries, the COVID-19 pandemic brought personal protective equipment (PPE) to the forefront of everyday life. In the United States, specialized respiratory protection (i.e., filtering facepiece respirators [FFRs], such as N95s) were reserved for high-risk occupations (i.e., healthcare) during supply shortages. Even with the redirection of supplies to healthcare, resources were still limited, which necessitated large-scale decontamination operations, primarily using hydrogen peroxide vapor systems [1–6]. In addition to FFRs, other types of PPE or non-specialized items such as cloth face coverings were used, and many times reused, by the general public. The general guidance by the Centers for Disease Control and Prevention (CDC) and other government agencies was to launder the face coverings in warm water. However, research into other effective small-scale disinfection or decontamination strategies was warranted to provide other options in addition to general laundering and/or natural attenuation, to minimize potential exposure to the SARS-CoV-2 virus when reused. The large-scale, commercial, specialized methods that may be appropriate for hospitals or laboratories may not translate for safe use by the average home or small business owner. Comprehensive data on how effective different small-scale or non-commercial disinfection strategies are for PPE and clothing items is limited in the literature. To help provide PPE/clothing disinfection efficacy data for the public, this study was conducted (using an enveloped bacteriophage Phi6 and a non-enveloped bacteriophage MS2) for simple, low-technology approaches on a variety of PPE materials representing respiratory, face/eye, and body protection.

While the range of PPE materials tested in the scientific literature is narrow, there are lessons to be learned from the large-scale decontamination and associated studies that were conducted for PPE in healthcare industries [1–5]. Large-scale decontamination was addressed by the CDC issuing FFR crisis capacity strategies such as limited reuse and decontamination to counteract the supply shortages [7]. In lieu of this public health emergency determination that authorized emergency use authorizations (EUAs), multiple healthcare decontamination systems were granted EUA approval through the Food and Drug Administration (FDA) [8]. Primary technologies for respirator decontamination involved vaporized hydrogen peroxide (VHP), ultraviolet-C radiation (UVC), and moist heat (steam) for virus inactivation. These EUAs have since been revoked, however, research on and use of these interim technologies provided important findings in the event of future pandemics or other public health emergencies. It is important to note that disposable FFRs should only be reused when operating at crisis capacity due to the inability of FFR supplies to meet the rate of demand [9].

The CDC/National Institute for Occupational Safety and Health (NIOSH) reviewed and summarized the decontamination technologies that had the most potential for use at the beginning of the pandemic [9]; multiple studies conducted prior to the COVID-19 pandemic that could be leveraged. Viscusi et al [10] evaluated five decontamination methods for FFRs; (UVC), ethylene oxide (EtO) and VHP were found to be the most promising decontamination methods. In 2015, Lindsley et al. [11] looked at the effects of UVC on N95 filtration performance and structural integrity and suggested that UVC may be effective for respirator disinfection and reuse, but the number of disinfection cycles before N95 degradation varied according to N95 model and dose. Additionally, three decontamination treatments (UVC, microwave-generated steam, and moist heat) for influenza virus (H5N1) on FFRs were evaluated by Lore et al. [12] in 2011. They found that all three treatments demonstrated effective decontamination of > 4-log reduction for N95s. More recently, Fischer et al. [2] evaluated four different decontamination methods, UV light, dry heat, 70% ethanol, and VHP, for their ability to reduce contamination of SARS-CoV-2 and their effect on N95 respirator function. They found that 70% ethanol affected mask filtration and performance, so it was not recommended. Due to effective virus inactivation and maintenance of respirator integrity, VHP was the highest recommendation, followed by UV and dry heat. Another study was published on the development of a low-cost VHP method for disinfecting PPE (coveralls, face shields, and N95s) in a small room (Saini et al., 2020) [13] and accounted for the integrity of the PPE following disinfection cycles. Additional studies on effective decontamination technologies for FFRs were reviewed, summarized, and published in 2020, which provided a snapshot of the existing literature on UVC and heat disinfection for FFR performance, fit and material integrity [14]. Most recently in 2021, an additional study focused on successful decontamination of N95s and face shields in a biosafety cabinet using the built-in UV light [15]. Results from these studies showed that UV, VHP and heat (moist and dry) were effective at disinfecting/decontaminating FFRs and other frequently used PPE items. The primary focus of most of these studies was on decontamination of healthcare respiratory protection as frontline workers during the pandemic rely on these devices to protect them from inhalation of pathogenic bioaerosols. However, these specialized methods suitable for hospitals or large laboratories are not typically accessible or designed to be used safely by the average home or small business owner.

The early studies that occurred prior to and early in the pandemic were essential in providing initial data to support large-scale disinfection/decontamination methods for healthcare. However, additional gaps remained for identifying and evaluating simple, low-tech, effective disinfection methods to be used by the general public and small businesses to disinfect PPE and clothing items, including those worn on the face, body, and feet.

In this research, bench-scale experiments were conducted using small coupons (swatches) of a large variety of PPE/barrier protection materials inoculated with virus and tested against a range of decontamination methods including bleach-, alcohol- and quaternary ammonium compound (QAC)-based liquid sprays, as well as a low concentration hydrogen peroxide vapor (LCHPV) technique tested by EPA [16]. Bench-scale laundering was also evaluated as a cleaning method that can be widely available for use in households. While these disinfection efficacy experiments were developed for use during the COVID-19 pandemic, they are also intended to provide a framework adaptable to other viral disinfection approaches for future pandemics. It should also be noted that face coverings and procedural masks are included in this study under “respiratory protection”, even though they are not technically considered respiratory protection PPE per OSHA, NIOSH or other government agencies. However, because they are worn over the nose and mouth and provide some level of reduction in inhaled viral concentration, they were included in the same category as N95s. The overall goal of this research is to provide information on the effectiveness of selected disinfection methods and commercial off-the-shelf (COTS) products for the cleaning and disinfection of specialized and non-specialized PPE items for potential reuse during a pandemic.

## Materials and methods

### Virus and bacteria

Experiments were performed using two viruses: the enveloped bacteriophage Phi6 with bacterial host *Pseudomonas syringae* and the non-enveloped bacteriophage MS2 (ATCC 15597-B1) with host *Escherichia coli* (ATCC 15597). Non-enveloped viruses demonstrate more germicidal resistance than non-enveloped viruses [17]. Phi6 and MS2 were propagated, prepared into stocks, and enumerated using the same methods and equipment described in Calfee et al. 2021 [18] and Monge et al. 2021 [19], respectively. In brief, a conventional soft agar overlay method [20] was used for both Phi6 and MS2 viruses. Method development was necessary to identify inoculum stabilizers that would provide a dynamic range large enough to accommodate the research goal of determining if a disinfectant could achieve a three-log reduction in viral load as necessary per EPA guidelines to label a potential product as virucidal [21]. Stabilizing agents were added to the inoculum and viruses were tested with stabilizing agents (10 μL pipetted) at two hours post inoculation on each PPE material at ambient conditions. Inoculum stabilization was achieved using 0.22 μm filter sterilized 10% beef extract (Sigma-Aldrich, Millipore Sigma, St. Louis, MO, USA; P/N B4888-100g) in phosphate-buffered saline (PBS) (Thermo Fisher Scientific, Waltham, MA, USA; P/N 08-757-100D) in the Phi6 inoculum and 0.22 μm filter sterilized 5% Fetal Bovine Serum (Gibco, Thermo Fisher Scientific, Waltham, MA, USA; P/N 10082139) in PBS in the MS2 inoculum. PPE and clothing test coupon inoculation is detailed in the PPE Materials subsection. Data from the method development tests are included in the Tables A and B in S1 Appendix.

Disinfection efficacy was calculated as the difference between the average log recovery of the positive controls and the average log recovery of the test coupon replicates.


$$Disinfection\;Efficacy=Average\;\log_{10}\left(Positive\;Controls\right)-Average\;\log_{10}\left(Test\;Coupons\right)$$ (Eq 1)

The detection limit for enumeration was 1 log plaque forming units (PFU) per sample. The upper limit of log reduction values was dependent upon the positive control recovery. When no virus was recovered from the test coupon, the disinfection efficacy was reported as the average positive control value minus the detection limit of 1 log PFU. The open-source statistical software R version 4.1.3 was used for the data analysis and figure generation.

### Disinfectants

**Fig 1:**
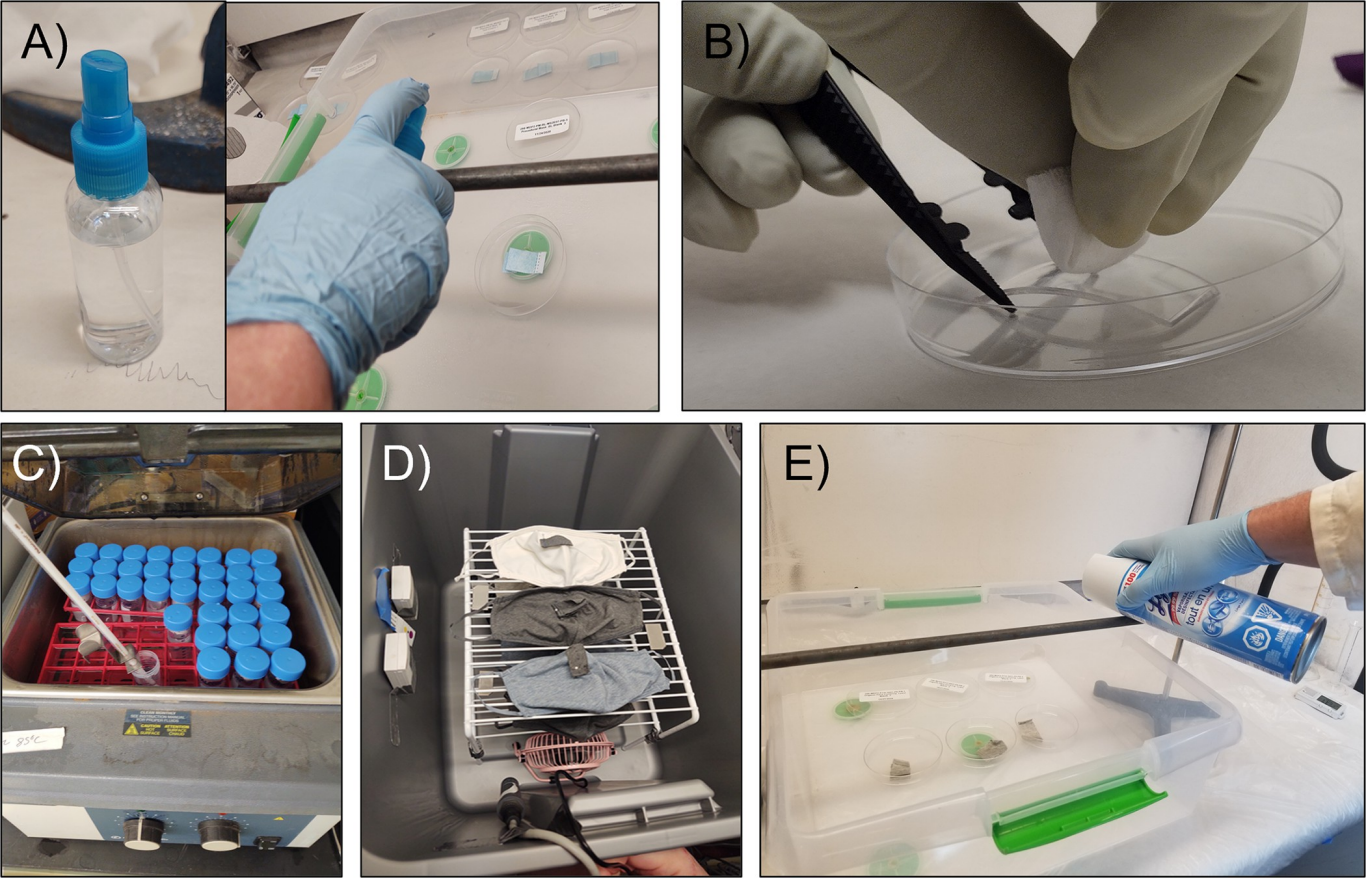
Disinfectant methods used in this research. A) bleach spray application using a mini-spray bottle, B) IPA wipe, C) bench-scale simulated laundering, D) LCHPV, and E) QAC application. Note, bars of fixed height were used in bleach and QAC application to standardize the application distance for each experiment (as depicted in Panels A and E).

Five cleaning/disinfection methods were tested: bleach spray, isopropyl alcohol (IPA) wipes, laundering (cleaning) using an aqueous solution of non-ionic low-sudsing detergent, LCHPV, and a QAC spray (Fig 1). To ensure contact times were maintained, tests were performed with each disinfectant that demonstrated that the use of 10% Dey-Engley (DE) neutralizing broth (Thermo Fisher Scientific, Waltham, MA, USA; P/N DF0819-17-2) during extraction quenched any residual disinfecting action during enumeration. The following individualized methods were used for application of each disinfectant:

#### Bleach

A diluted bleach solution (The Clorox Company, Oakland, CA, USA; P/N Clorox® Regular-Bleach) was prepared using the U.S. Center for Disease Control’s (CDC’s) recommended recipe of 80 mL household bleach (5.25–7.5% sodium hypochlorite) in 3.8 L, which is equivalent to 1,000–1,500 ppm sodium hypochlorite. Free available chlorine (FAC) was also determined prior to each test by measurement with analysis equipment (Hach® high-range bleach test kit, Method 10100 [model CN-HRDT]) and the validity of the FAC measurements were confirmed through the titration of a chlorite ion standard. The diluted bleach was spray-applied (Fig 1A) using a 29.6 mL spray bottle with a spray rate of 9 mL/min. The applied spray amount was gravimetrically determined to range from 0.42–0.50 grams of solution per sample and allowed 30 minutes of contact time prior to neutralization during extraction. Because multiple PPE items tested were porous and non-absorbent, this longer contact time was chosen for disinfectant cycles of all PPE materials.

#### IPA

Each inoculated coupon that had dried was placed in a sterile petri dish and wiped four times with ¼ of a pre-cut 70% IPA wipe (CVS Pharmacy, Woonsocket, RI, USA; P/N 444797). The surface of each coupon was wiped two times (top to bottom), then the wipe was folded, and the surface of the coupon was wiped from top to bottom an additional two times (Fig 1B). The coupons then sat for 30 minutes, uncovered at ambient temperature prior to extraction.

#### Laundering

To simulate washing and drying on a bench scale, a previous method developed by EPA for bacterial spores was used [22]. Coupons were placed in 50 mL polypropylene conical tubes (FALCON A Corning Brand, REF 352098) filled with 40 mL of a 0.4% (by volume) sterilized tap water mixture of Tide® Original Scent Liquid Laundry Detergent that had been warmed to 50°C (Proctor and Gamble, Cincinnati, OH, USA; P/N 003700023068). The tubes were placed in an orbital shaker fitted with a platform designed to hold 50 mL conical tubes and shook at 250 rpm at ambient temperature for a 9-minute wash cycle (Fig 1C). To simulate a rinse cycle, coupons were transferred to a fresh conical tube containing 40 mL of sterile tap water at ambient temperature, returned to the orbital shaker, and shook at 250 rpm at ambient temperature for another 9-minute cycle. At the completion of the rinse cycle, the coupons were aseptically removed from the conical tube, the rinse water disposed of, and a hair dryer on warm setting was used to dry the coupon until visually no longer wet. The coupon was then returned to the tube and extracted.

#### LCHPV

The LCHPV setup is shown in Fig 1D. A small plastic bin with lid (Sterilite, Townsend, MA, USA, P/N 002-02-0479) was outfitted with a metal storage rack (Smart Design® Pro-mart, Irvine, CA, USA; P/N 8233188), a 4-inch metal mini-360-degree pivot USB personal desktop fan (Walmart, Bentonville, AR, USA P/N 577623661), HOBO temperature and relative humidity data loggers (Onset Computer Corporation, Bourne, MA, USA; P/N U10-003), a hydrogen peroxide chemical indicator strip (Sterafirm-STERIS, Mentor, OH, USA; P/N PCCO60), and an electrochemical sensor capable of detecting 0–25 ppm hydrogen peroxide (Analytical Technology, Inc. Collegeville, PA, USA, Model B12–34–5–0025–1). 237 mL of a 3% liquid hydrogen peroxide solution (3% U.S.P Hydrogen Peroxide, Aaron Industries Corp, Leominster, MA, USA; P/N 665214) was placed in a plastic container below the metal storage rack and tested with peroxide test strips, dipped in the liquid, prior to insertion for concentration quality control (Indigo Instruments, Waterloo, ON, Canada; P/N 33815-P10 pc). Based on the vapor pressure of hydrogen peroxide, a vapor is generated inside the closed container and is a function of temperature. Three test replicates of full-size PPE (face covering or procedural mask) with an inoculated PPE coupon and a chemical indicator strip attached to their surfaces (Fig 1D) were placed on the rack along with a procedural blank and additional PPE items (2 additional for face coverings or 8 additional for procedural masks) that were included in the bin to accurately account for the material demand of the hydrogen peroxide, should the testing apparatus be utilized to capacity. Inoculated stainless steel controls were also added inside the bin during each test; face coverings and procedural masks were tested separately for Phi6 and MS2. Samples were disinfected over an 18-hour disinfection cycle. Temperature, relative humidity, and peroxide concentration data from the experiments are included in the Figs A-D in S2 Appendix. For face covering tests, the average hydrogen peroxide concentrations during the tests were 4.7 ppm during the MS2 experiment and 3.95 ppm for the Phi6 experiment. For the procedural masks, the average hydrogen peroxide concentration during the tests were 10.6 ppm during the MS2 experiment and 8.7–11.7 ppm (depending on the use of pre or post-test sensor calibrations, respectively) for the Phi6 experiment. Larger variation in the hydrogen peroxide sensor readings were observed during the Phi6 experiment, so the average range with and without post-calibration correction is reported.

#### QAC

Lysol^®^ disinfectant spray (active ingredients: benzalkonium chloride and ethanol) was applied using the manufacturer’s container (Reckitt Benckiser LLC, Parsippany, NJ, USA; P/N 1920074186). Coupons were sprayed for a three second duration per three coupons and left for a disinfectant-inoculated coupon contact time of 30 minutes in an uncovered petri dish prior to extraction (Fig 1E). A gravimetric spray amount delivered over three triplicate coupons during each test was measured and an average of 0.45 +/- 0.04 grams was calculated per single coupon sample.

### PPE materials

**Fig 2:**
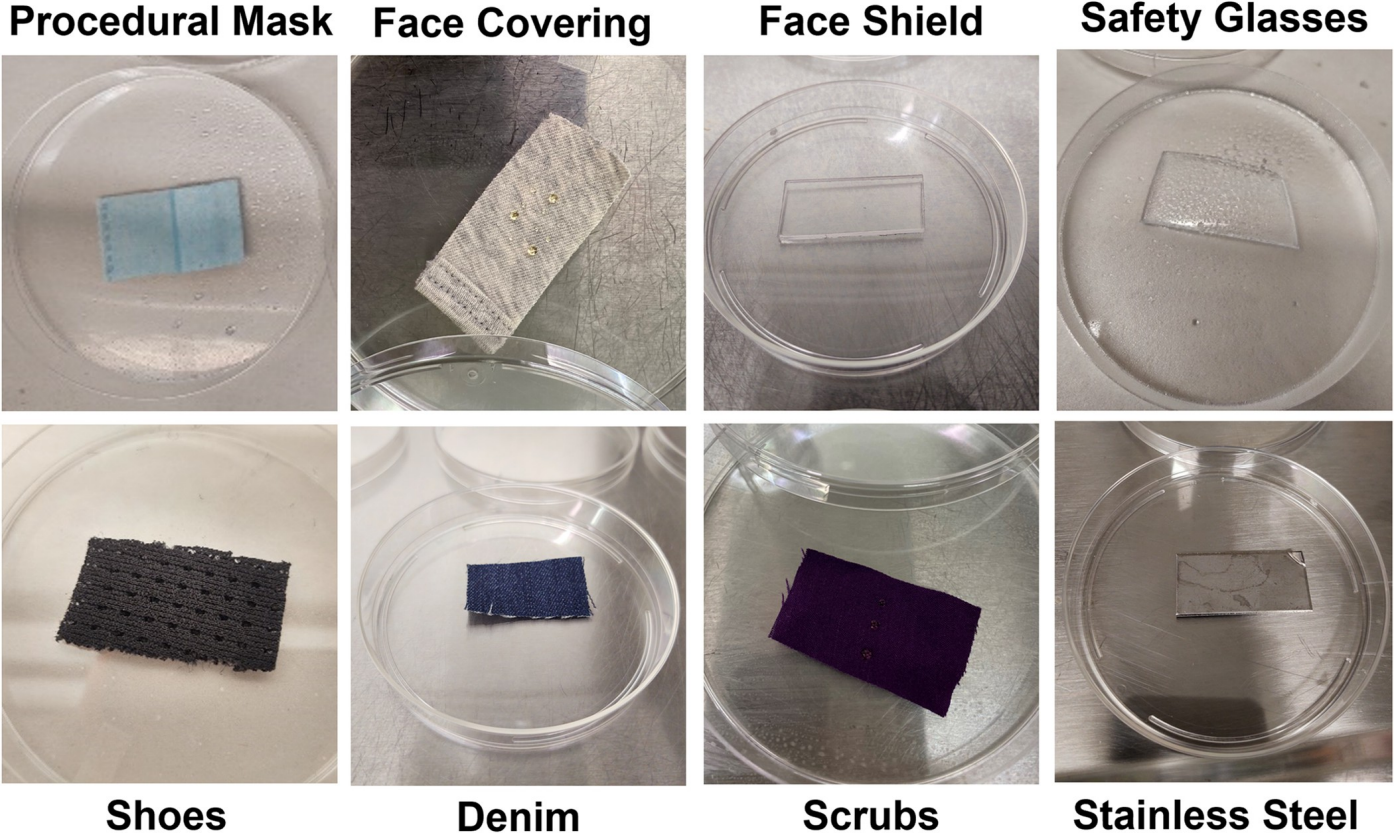
PPE test materials. Coupons of each PPE type (procedural mask, face covering, face shield, safety glasses, shoes, denim, scrubs, and stainless steel) that were used in tests.

Small 2 x 4 cm coupons were cut from full-sized PPE and clothing items and are shown in Fig 2. Most coupons were inoculated in triplicate (3 coupons per PPE material) with 10 μL of 2 x 10^9^ PFU/mL virus solution for a target of 2 x 10^7^ PFU/coupon. Seven different types of PPE and clothing items (Table 1) were tested. A subset of Phi6 coupon-disinfectant combinations (face shield-IPA, face shield-QAC, procedural mask-IPA, procedural mask-QAC, safety glasses-IPA, shoes-IPA, and shoes-QAC) were inoculated at 2 x 10^6^ PFU/coupon during initial experiments and were not repeated at the higher inoculation titer because virus was still detectable post-disinfection, and disinfection efficacy could be calculated. The PPE materials required different drying times after the inoculation spike determined by visual inspection: 120 minutes for procedural masks, safety glasses, stainless steel, and face shields, 60 minutes for face covering, and 30 minutes for shoes, denim, and scrubs. A set of procedural blanks (not inoculated, but disinfected), controls (inoculated, but not disinfected), and stainless-steel reference coupons were included in each experimental run for quality assurance and quality control. PPE materials were not tested with all disinfects. Combinations were selected based on known material compatibility, practicality of the disinfection method, and budget (Table 1).

**Table 1:** Test matrix for experiments conducted herein. An “X” denotes that a given combination of PPE or clothing material was tested against a particular cleaning or disinfection method.

PPE Item	Disinfection method				
	LCHPV	Laundering	Bleach	IPA	QAC
***Non-specialized Respiratory Protection***					
Face covering	X	X	X	X	X
*Felina Reusable Organic Cotton Face Masks*, *PN*: *990121P4*, *Chatsworth*, *CA*, *USA*					
Procedural mask	X		X	X	X
*3M Earloop Procedure Face Mask 1820*, *St*. *Paul*, *MN*, *USA*					
***Face and Eye Protection***					
Face shield			X	X	X
*Suncast Commercial PPE Face Shield for Healthcare Use*, *PN*: *HGASSY16*, *Batavia*, *IL*, *USA*					
Safety glasses			X	X	X
*Pyramex Intruder Safety Glasses with Clear Uncoated Lens*, *PN*: *WS19100PT*, *Piperton*, *TN*, *USA*					
***Non-specialized Body Protection***					
Medical scrubs		X			
*Gogreen cool*, *PN*: *WS19100PT*, *Charlotte*, *NC*, *USA*					
Denim		X			
*Wrangler Rustler Regular Fit Straight Leg Jean Four Pocket Jean with Scoop Front Pockets*, *PN*:*87619PW*, *Greensboro*, *NC*, *USA*					
Shoes		X	X	X	X
*Athletic Works Men’s Runner Athletic Shoes*, *PN*: *577021790*, *Walmart*, *Bentonville*, *AR*, *USA*					

## Results

**Fig 3:**
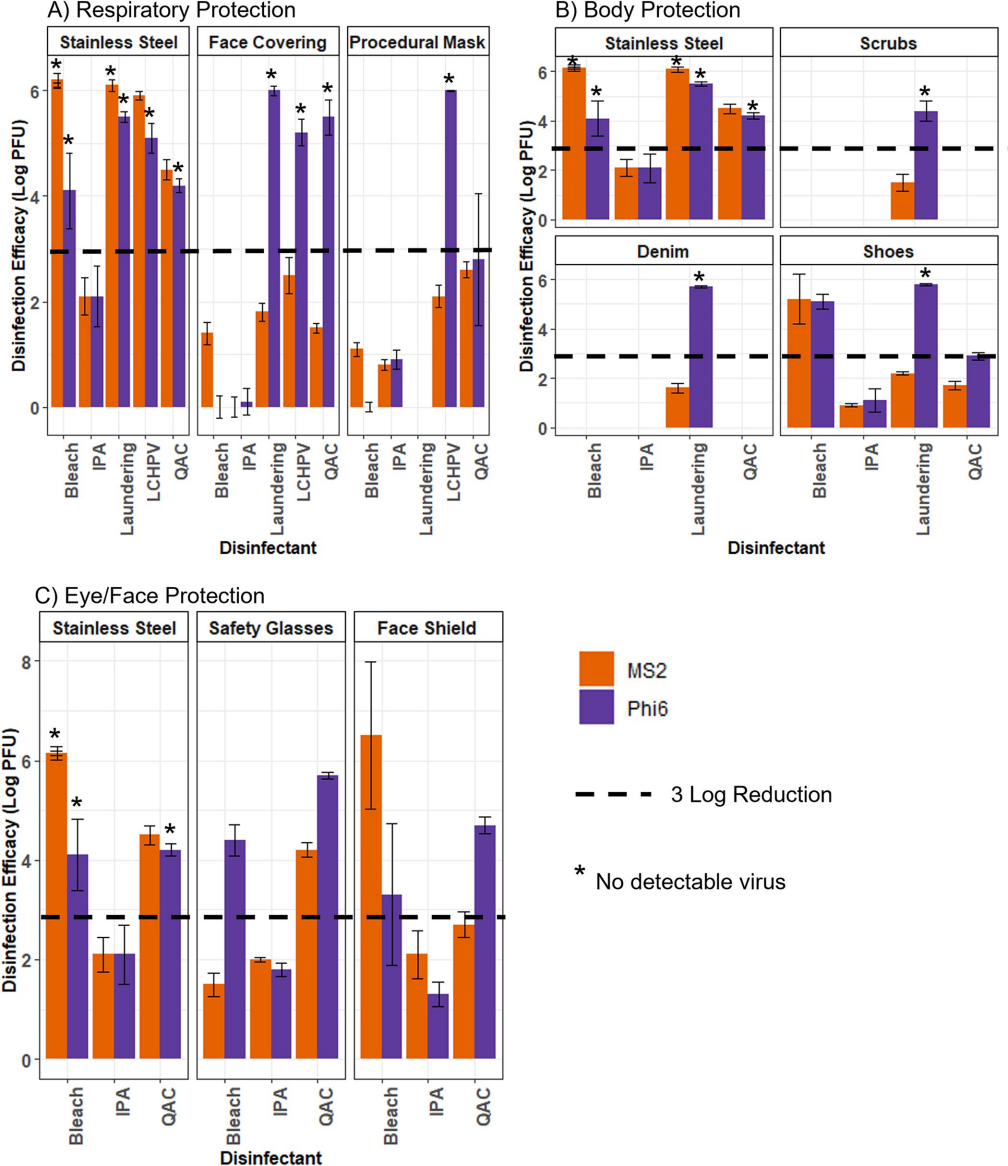
Disinfection efficacy results in log PFU by disinfectant for stainless-steel control coupons and A) respiratory protection (face covering and procedural mask coupons), B) body protection (scrubs, denim, and shoe coupons), and C) eye/face protection (safety glass and face shield coupons). The dashed line on each bar chart marks the 3-log reduction virus disinfection criteria, and a * above a bar indicates that no virus was detected on the test coupon. Error bars are the pooled standard deviation of the test coupons and positive control coupons. Where no virus detected is notated, but an error bar is present it represents the variability in the positive controls. Stainless steel data also averages data from multiple tests. Where no virus detected is notated for stainless steel it is applicable to all test coupons.

Fig 3A contains the log PFU disinfection efficacy results for PPE items used for respiratory protection (face coverings and procedural masks). This study uses a benchmark based on EPA’s current Product Performance Test Guideline 810.2200, for both materials and viruses tested. The product should demonstrate a ≥ 3-log reduction on every surface in the presence or absence of cytotoxicity.

IPA failed to achieve a 3-log reduction. Further, none of the disinfectants achieved 3-log reduction in viral load for MS2-contaminated respiratory protection. LCHPV was effective (≥ 3-log reduction) for both face coverings and procedural masks for Phi6. The QAC disinfectant achieved 3-log reduction for Phi6 for face coverings but not for the procedural mask. Laundering was only tested on the face coverings and was effective (≥ 3-log reduction) at Phi6 disinfection for this material.

Fig 3B contains the disinfection efficacy results for PPE items used for body protection (scrubs, denim, and shoes). IPA was only tested on shoe coupons but failed to achieve 3-log reduction for this material. A 3-log MS2 disinfection efficacy was not achieved for the scrubs and denim coupons (only laundering was tested as a disinfection method) but was achieved for the shoes using the diluted bleach solution.

Fig 3C contains the disinfection efficacy results for PPE items used for eye and face protection (safety glasses and face shields). QAC achieved a 3-log reduction of MS2 for safety glasses. Bleach achieved a 3-log reduction of MS2 for face shields. For Phi6, both bleach and QAC were effective on both safety glasses and face shields.

**Fig 4:**
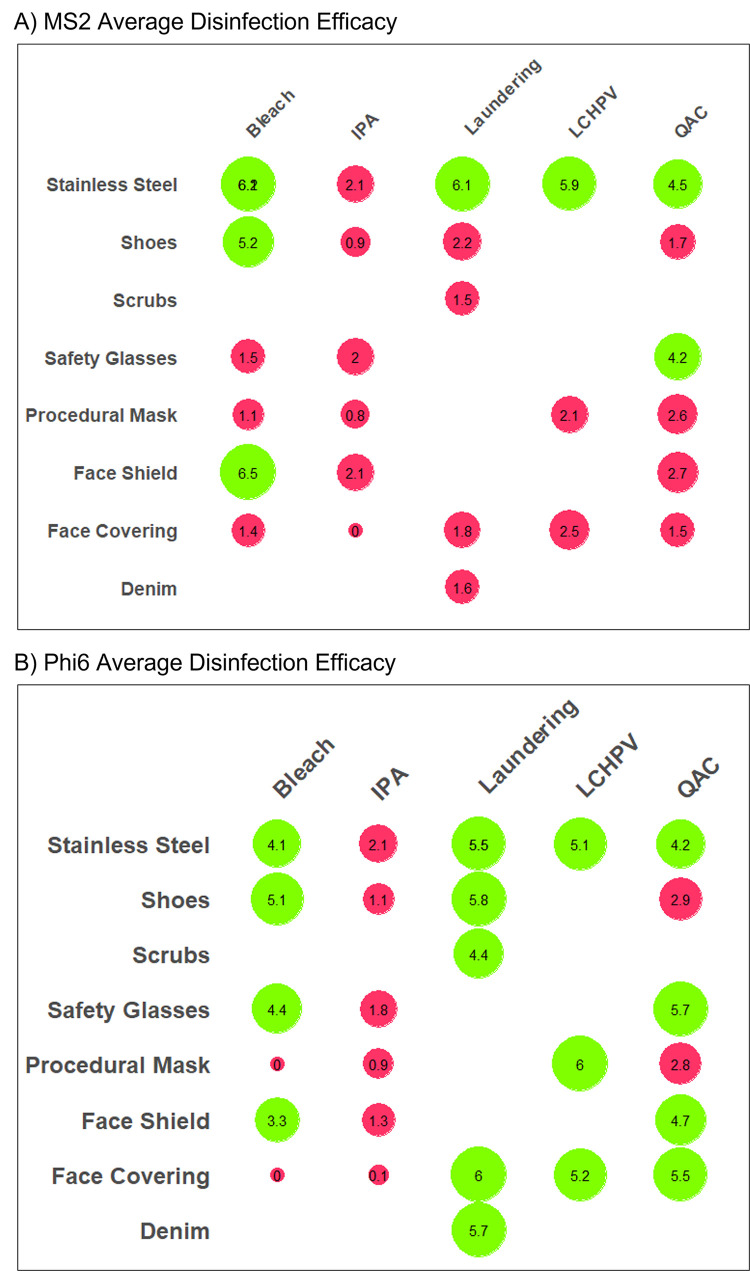
Average disinfection efficacy of each disinfectant and material for A) MS2 and B) Phi6 with red shading if less than the 3-log disinfection efficacy metric and green if greater than 3-log. Size of bubbles is reflective of efficacy. Larger size indicates a higher Log PFU removal by the disinfectant.

Overall, disinfectants were less efficacious against MS2 than Phi6 (Fig 4). IPA wipes did not achieve a 3-log reduction for either virus on any of the materials. Aside from IPA, all of the disinfectants achieved the target efficacy on the stainless-steel coupons for both types of viruses. Bleach provided effective disinfection for shoes and face shields for both types of viruses, as well as safety glasses for Phi6. Laundering and LCHPV were effective (≥ 3-log reduction) for all PPE items tested for Phi6, and none of the items tested for MS2. QAC was effective for both virus types for safety glasses and additionally for face shield and face coverings for Phi6.

## Discussion

During the initial stages of the COVID-19 pandemic, there were many unknowns regarding transmission of the SARS-CoV-2 virus and what kinds of protective measures would be most effective for minimizing exposure. Because of these unanswered questions, research focused on the highest need areas, which were those in high-risk occupations like healthcare, due to their higher relative exposure risk. Because of this priority to address PPE shortages for healthcare workers, larger scale disinfection/decontamination methods for disposable respirators (FFRs) were evaluated in a rapid timeframe during the need for crisis capacity strategies. Many scientific publications focusing on NIOSH-recommended decontamination technologies for FFRs including UVC [2, 15], hydrogen peroxide [1–5], dry heat [2, 4], and moist heat [4] were produced in an unprecedented short period of time. However, gaps still exist for research on simple, low-cost disinfection methods.

This research sought to ascertain simple, effective disinfection methods that could be employed by most anyone to clean/disinfect their PPE and clothing items on a small scale using common disinfectants. SARS-CoV-2 was not used in this study, but the use of Phi6 and MS2 allowed for a more cost-effective option with a larger set of test conditions. However, the use of an enveloped virus (Phi6) and non-enveloped virus (MS2) bounded the effort in terms of the range of viral resistance to disinfectants to allow the application of these findings not only to the current COVID-19 pandemic but also contagion outbreaks as well.

In this coupon study, most disinfection methods used had varying results between the two viruses. As expected, the enveloped virus Phi6, was overall easier to inactivate than MS2. This was expected as MS2 has greater germicidal resistance due to the lack of an envelope (lipid membrane) [23, 24]. Each disinfection method and its efficacy is discussed in more detail below.

### Bleach

Diluted bleach disinfection spray was tested for efficacy on face coverings, procedural masks, safety glasses, face shields and shoes. Efficacy varied for these PPE items, as it was not effective on Phi6 or MS2 for the porous respiratory protection items but was effective on both viruses for shoes and face shields. Efficacy was mixed for Phi6 and MS2 on safety glasses, with Phi6 being easier to inactivate. Overall, bleach spray may have application for specific PPE/clothing items that are non-porous. Application on shoes may be effective, but it may also degrade shoes over time and repeated applications. Additionally, bleach use on items worn over the nose and mouth would not be encouraged due to the lingering bleach odor and potential material degradation.

### IPA

IPA was used as a disinfectant in these tests as a commercial off-the-shelf wetted wipe, which limited its effectiveness, as alcohol-based products are expected to be efficacious against especially enveloped viruses but were not in this study. It was noted during testing that the wipes were lightly wetted and did not provide enough disinfectant to adequately cover the surface with liquid, and when used on porous items, efficacy was low for Phi6 and MS2. IPA did not demonstrate efficacy greater than 3-log reduction for a single PPE/clothing item, likely due to low liquid volume per virus surface concentration. As such, IPA wipes were not shown to be effective even for non-porous safety glasses and face shields. Liquid spray of IPA was not tested due to flammability concerns of the alcohol.

### Laundering

Laundering cleaning/disinfection efficacy has not been a major focus in the recent scientific literature. More commonly, studies have evaluated performance of cloth masks or face coverings following repeated laundry cycles [15]. In this study, the main focus was on efficacy and the laundering of PPE/clothing coupons was shown to be effective for all items inoculated with Phi6 (face coverings, scrubs, denim and shoes). Even though some efficacy (1-2-log reduction) was demonstrated for items inoculated with MS2, none of the items demonstrated ≥ 3-log reduction after laundering. This, again, highlights the difference in disinfection efficacy between an enveloped and non-enveloped virus, as Phi6 is less resistant to disinfectants. Laundering combines the physical action of the item in the liquid with potential viral inactivation from the detergent and the heat from drying. The simulated laundering of coupons was very effective (full inactivation) for Phi6, but not MS2. Laundering may be more effective against MS2 with the addition of a disinfectant like bleach.

These results may have implications for the effectiveness of laundering of clothing items (including face coverings) at home as it was shown that laundering can result in a range of efficacies, depending on the virus type. Factors such as water temperature, drying time and temperature and virus type should all be considered. However, since efficacy for MS2 was lower for coupon laundering, additional research on laundering protocols and additives such as bleach, which has shown to be effective on bacterial spores [25], is needed.

### LCHPV

The LCHPV method is a very simple disinfection method using off-the-shelf liquid hydrogen peroxide in an enclosed container to create a hydrogen peroxide vapor that PPE/clothing items can be exposed to over time. HPV decontamination has been widely studied and shown to be effective at higher concentrations using units such as the Bioquell unit that was granted an EUA for large scale healthcare disinfection of masks and respirators [8, 14]. However, there is little data on efficacy of this simple disinfection method using easily accessible off-the-shelf hydrogen peroxide and materials. One recent study by Saini et al. [13] investigated efficacy of a 7–8% VHP on three biological indicators including spiked *E*. *coli* and *M*. *smegmatis* onto coveralls, face shields and N95 masks. However, this study utilized room disinfection, which would not be suitable for the general public.

For this method, the coupons were attached to face coverings or procedural masks to simulate the material demand from disinfecting multiple masks at once. For Phi6, LCHPV was effective with ≥ 3-log reduction and achieving full disinfection for both respiratory protection items. For MS2, disinfection efficacy was lower and between 2-3-log reduction for both items. The same protocol was used for both viruses, but the concentration-time (ppm hours) used was not sufficient for MS2 as tested. As noted previously, lower concentrations of hydrogen peroxide vapor were measured during the face covering experiments (roughly half of procedural mask experiments), likely due to increased material demand by the cotton material. Further modifications to the LCHPV protocol (e.g., longer dwell time) may be needed to achieve the target efficacy goal for MS2, however, this is a simple method that could be employed by most anyone. For safety purposes, it is advised to set up the LCHPV system in a well-ventilated area or outside.

### QAC

The COTS QAC spray was tested on respiratory protection (i.e., face coverings), shoes, and eye/face protection items. Because this COTS product is readily available and easy to use, it was considered for testing on respiratory protection items. Even though it demonstrated good efficacy for Phi6 on face coverings, the residual odor from the QAC may preclude use of this disinfection technique for items worn over the nose and mouth. A period of time following spray disinfection would be necessary to demonstrate sufficiently low levels of QAC on the face covering, and this was not measured in our study. Therefore, using the QAC spray on full-size face coverings may not be warranted if other effective cleaning/disinfection methods exist.

Multiple COTS disinfectants including bleach, IPA, LCHPV and a QAC, were evaluated for their disinfection efficacy on PPE/clothing materials in this study. Under the Federal Insecticide, Fungicide, and Rodenticide Act (FIFRA), a pesticide must be registered by EPA before it can be legally sold or distributed in the United States. Once registered, pesticides must be used in a manner consistent with the approved label directions and claims. The results of this research do not supplant data required for product registration nor for adding additional claims to product labels. Products must be used in accordance with their label claims under FIFRA. EPA does not endorse the use of any products tested in this study.

This coupon efficacy study demonstrated repeatable liquid inoculation procedures and good recovery of non-pathogenic viruses (Phi6 and MS2 bacteriophages) from a variety of porous and non-porous PPE/clothing materials as evidenced by the positive control coupons. In order to ensure that each test condition had a sufficiently high dynamic range to demonstrate a 3-log reduction, high viral surface concentrations were used and are likely higher than would be encountered under real-world conditions. The cleaning and disinfection methods evaluated in these bench-scale tests that did not achieve a 3-log reduction may still provide some benefit in reducing the virus concentration, potential exposure and ultimately disease transmission, although this is dependent on a number of factors including PPE material, infectious dose, and environmental conditions. Multiple disinfection methods were shown to be effective in reducing viral loads from PPE coupons, though only laundering and LCHPV were effective for all materials tested for Phi6. In general, non-porous items were easier to disinfect than porous items. This was demonstrated by the stainless-steel control coupons (> 3-log reduction) for all disinfectants, except for IPA wipes. Several effective disinfection technologies were promising (laundering, LCHPV, and QAC) for specific materials and were selected to be included in efficacy testing with full-scale PPE/clothing items, which is the focus of ongoing research. This coupon study provides important data that can be used during a pandemic when crisis capacity strategies are warranted and people are in need of guidance on cleaning/disinfection of their commonly worn PPE/clothing items. However, it should also be noted that this study focused on biological agent inactivation on material coupons within a laboratory setting that integrated appropriate exposure controls. This study does not address the operational and health and safety requirements for use outside of the laboratory conditions in which the study was conducted.

## Supporting information

S1 Appendix Stability data. (DOCX)

S2 Appendix LCHPV environmental condition data. (DOCX)
