# Supplementary material for: Evaluation of disinfection methods for personal protective equipment (PPE) items for reuse during a pandemic
Source: PLoS One. 2023 Jul 27;18(7):e0287664. doi: 10.1371/journal.pone.0287664 (PMC10374148; doi:10.1371/journal.pone.0287664)
Supplement: S1 Appendix — (DOCX) [file pone.0287664.s001.docx]

**Supporting Information**

**S1 Appendix. Stability Data**

**Table A.** Phi6 Stability Data (10% Beef Extract)

*Procedural Blank Samples at or below detection limit

|  | **Average of Final Log PFU/Sample** | **Std Dev of Final Log PFU/Sample** | **Average of Final PFU/Sample** | **Std Dev of Final PFU/Sample** |
| --- | --- | --- | --- | --- |
| Inoculation Control-Procedural Mask, Denim | 6.7 | 0.04 | 4.7E+06 | 4.4E+05 |
| Inoculation Control – Scrubs | 7.1 | 0.03 | 1.4E+07 | 8.5E+05 |
| Inoculation Control - Others | 7.2 | 0.02 | 1.5E+07 | 8.7E+05 |
| **Inoculated PPE Samples** | | | | |
|  | | | | |
| Procedural Mask (120 min) | 6.5 | 0.05 | 3.1E+06 | 3.8E+06 |
| Denim (120 min) | 5.0 | 0.05 | 9.4E+04 | 1.0E+04 |
| Scrubs (30 min) | 5.4 | 0.58 | 4.3E+05 | 4.0E+05 |
| Face Covering (60 min) | 6.4 | 0.11 | 2.7E+06 | 6.8E+05 |
| Safety Glasses (120 min) | 6.4 | 0.34 | 2.9E+06 | 1.7E+06 |
| Shoes (30 min) | 6.7 | 0.08 | 4.7E+06 | 8.3E+05 |
| Stainless Steel (120 min) | 5.5 | 0.47 | 4.2E+05 | 3.7E+05 |

**Table B.** MS2 Stability Data (5% Fetal Bovine Serum)

*Procedural Blank Samples at or below detection limit

|  | **Average of Final Log PFU/Sample** | **Std Dev of Final Log PFU/Sample** | **Average of Final PFU/Sample** | **Std Dev of Final PFU/Sample** |
| --- | --- | --- | --- | --- |
| Inoculation Control | 7.8 | 0.07 | 6.3E+07 | 1.1E+07 |
| **Inoculated PPE Samples** | | | | |
| **0.5 Hours** |  |  |  |  |
| Denim | 7.3 | 0.02 | 2.1E+07 | 1.1E+06 |
| Scrubs | 7.2 | 0.17 | 1.5E+07 | 5.8E+06 |
| Shoes | 7.3 | 0.15 | 2.0E+07 | 6.4E+06 |
| **2 Hours** |  |  |  |  |
| Face Covering | 7.5 | 0.03 | 3.2E+07 | 2.5E+06 |
| Face Shield | 7.2 | 0.07 | 1.8E+07 | 2.8E+06 |
| Procedural Mask | 7.3 | 0.07 | 2.0E+07 | 3.2E+06 |
| Safety Glasses | 7.1 | 0.06 | 1.4E+07 | 1.9E+06 |
| Stainless Steel | 7.1 | 0.52 | 1.7E+07 | 1.2E+07 |
| **18 Hours** |  |  |  |  |
| Face Covering | 7.1 | 0.47 | 1.8E+07 | 1.3E+07 |
| Face Shield | 7.1 | 0.05 | 1.2E+07 | 1.6E+06 |
| Procedural Mask | 6.9 | 0.07 | 8.1E+06 | 1.2E+06 |
| Safety Glasses | 7.3 | 0.08 | 2.0E+07 | 3.7E+06 |
| Shoes | 6.6 | 0.11 | 3.9E+06 | 9.6E+05 |
| Stainless Steel | 6.6 | 0.06 | 3.7E+06 | 5.0E+05 |
