# Supplementary material for: Evaluation of disinfection methods for personal protective equipment (PPE) items for reuse during a pandemic
Source: PLoS One. 2023 Jul 27;18(7):e0287664. doi: 10.1371/journal.pone.0287664 (PMC10374148; doi:10.1371/journal.pone.0287664)
Supplement: S2 Appendix — (DOCX) [file pone.0287664.s002.docx]

**Supporting Information**

**S2 Appendix. LCHPV Environmental Condition Data**

**Figure A. Phi6 Experiments Face Coverings**

**Figure B. Phi6 Experiments Procedural Masks**

**Figure C. MS2 Experiments Face Coverings**

**Figure D. MS2 Experiments Procedural Masks**
